# Perspectives of patients and clinicians on older patient mobility on acute medical wards: a qualitative study

**DOI:** 10.1186/s12877-023-04226-0

**Published:** 2023-09-13

**Authors:** Philippe J. Herzog, Rose D. L. Herzog-Zibi, Martina Mattmann, Charlotte Möri, Blandine Mooser, Jennifer Inauen, Carole E. Aubert

**Affiliations:** 1 Department of General Internal Medicine, Inselspital, Bern University Hospital, University of Bern, Bern, Switzerland; 2 Institute of Psychology, University of Bern, Bern, Switzerland; 3 Institute of Primary Health Care (BIHAM), University of Bern, Bern, Switzerland

**Keywords:** Hospital mobility, Mobilization, Medical ward, Qualitative, Perspectives, Barriers, Facilitators

## Abstract

**Background:**

Low mobility during an acute care medical hospitalization is frequent and associated with adverse outcomes, particularly among older patients. Better understanding barriers and facilitators to improve mobility during hospitalization could help develop effective interventions. The goal of this study was to assess barriers and facilitators to older medical patients’ hospital mobility, from the point of view of patients and clinicians, to develop a framework applicable in clinical practice.

**Methods:**

We conducted a qualitative study in one university and two non-university hospitals of two different language and cultural regions of Switzerland, including 13 focus groups (FGs; five with patients, eight with clinicians). We included 24 adults aged 60 years or older hospitalized on an acute general internal medicine ward of one of the three participating hospitals during the previous years, and 34 clinicians (15 physicians, nine nurses/nursing assistants, 10 physiotherapists) working on those wards. The FG guides included open-ended questions exploring mobility experiences, expectations, barriers and facilitators to mobility, consequences of low mobility and knowledge on mobility. We applied an inductive thematic analysis.

**Results:**

We identified four themes of barriers and facilitators to mobility: 1) patient-related factors; 2) clinician-related factors; 3) social interactions; and 4) non-human factors. Clinician-related factors were only mentioned in clinician FGs. Otherwise, subthemes identified from patient and clinician FGs were similar and codes broadly overlapped. Subthemes included motivation, knowledge, expectations, mental and physical state (theme 1); process, knowledge – skills, mental state – motivation (theme 2); interpersonal relationships, support (theme 3); hospital setting – organization (theme 4).

**Conclusions:**

From patients’ and clinicians’ perspectives, a broad spectrum of human and structural factors influences mobility of older patients hospitalized on an acute general internal medicine ward. New factors included privacy issues and role perception. Many of those factors are potentially actionable without additional staff resources. This study is a first step in participatory research to improve mobility of older medical inpatients.

**Supplementary Information:**

The online version contains supplementary material available at 10.1186/s12877-023-04226-0.

## Introduction

Mobility, defined as any kind of movement (from in-bed movement to walking up the steps), is frequently limited during an acute care hospitalization, which is associated with adverse outcomes [1–5]. This can result in disability, institutionalization and death [1–5]. Low mobility during a hospitalization is particularly common in older patients, who are also more vulnerable to its adverse outcomes and less likely to recover [2, 6]. After one year, only 30% of patients who experienced functional decline during hospitalization have recovered and 40% have died, compared to 18% of those without functional decline [7].

Unfortunately, interventions that succeeded to improve hospital mobility in strict study conditions have not been implemented in clinical practice on a large scale [5, 8, 9]. This might be due to a lack of comprehensive consideration of barriers, facilitators, and real-life resources, and of active stakeholder involvement (“participatory research”) [10]. To implement long-term changes, we need to address context- and population-specific barriers and facilitators using available resources.

Assessing and integrating stakeholder perspectives is the first step towards participatory development of scalable interventions [10]. Several themes influencing mobility during an acute hospitalization on a medical ward have been identified in previous research, including patient situation, knowledge, beliefs, experiences, intentions, emotions, social influences, role/identity, implementation/organization and environment/resources [11–13]. However, most studies focused on one or a few number of stakeholder categories (e.g., nurses and patients), while we lack an integration of the perspectives of the different stakeholders involved (including the patients, physicians, nurses, nursing assistants and physiotherapists) in a framework that could be directly applied in clinical practice to improve mobility of older hospitalized medical patients. Furthermore, many studies did not focus on older medical inpatients, for which barriers and facilitators might differ from other populations.

The goal of this study was therefore to assess barriers and facilitators to mobility of older patients hospitalized on an acute medical ward from the point of view of patients and clinicians, and to integrate their perspectives in a framework that can be directly applied in clinical practice to address barriers and facilitators to mobility of older adults acutely hospitalized on a medical ward.

## Methods

### Research team

The research team included one attending physician working at Bern University Hospital (CA, female), two general practitioners working in an ambulatory care practice (RH, female, and PH, male), two students completing their final year of a master in health and behavior psychology (MM and CM, both females), and a professor of psychology working at the University of Bern (JI, female). CA, MM, CM and JI were specifically trained in qualitative studies.

### Design

We used a case study qualitative design with focus groups (FGs) to assess perspectives on hospital mobility of older medical patients. A case study approach was chosen because we wanted to conduct an in-depth investigation of mobility among key stakeholders involved in patient mobility [14]. Data are reported according to the COREQ checklist.

### Setting

The study was conducted between March and April 2022 in the following three hospitals from German- and French-speaking regions of Switzerland: 1) Bern University Hospital (Inselspital), a large university hospital in Bern, 2) Tiefenau Hospital, a small non-university hospital in Bern, and 3) Fribourg Cantonal Hospital (HFR-Fribourg), a large non-university hospital in Fribourg. To broaden generalizability, the sites were selected to represent a variety of hospital sizes/types and language/cultural regions. The duration of patient and clinician FGs was 90 and 60 min, respectively. We planned more time for patient FGs based on past experience.

### Participants and sample size

All participants were informed that their data would be treated anonymously and their name appear nowhere. They were also asked not to share the content discussed during the FG with other persons. At the beginning of the FG, the goal and reasons of the research were explained, and the research team was presented to the participants.

#### Sample size

We estimated that five FGs with patients and nine FGs with clinicians with four to five participants in each group (details below) would allow to reach data saturation. A higher number of FGs with clinicians was planned because we wanted to conduct FGs both with individual categories of clinicians (e.g., only physicians) and with a mix of professions (see following paragraph on Clinicians). We expected that we would reach data saturation with this number of participants. However, since we could not be sure that it would be the case, we decided that we would conduct additional FGs if we noticed during data analysis that this was not the case. The small number of participants per FG was chosen to allow enough time and opportunities to all participants to express their perspectives.

#### Patients

Patient recruitment was done by phone by two authors (RH, PH) based on a list of patients aged 60 years or older, hospitalized in a medical ward of one of the participating hospitals in the previous year. The list was provided by the hospitals after receiving the answer of the ethical committee. Exclusion criteria were: dementia and incapacity to walk. After checking inclusion criteria, patients were called in alphabetical order by taking the most recent hospitalizations. Patients who could not be reached after three call attempts on different days and times were excluded. We planned five FGs with five patients each, but recruited six patients per group to account for last-minute withdrawal. Recruitment was stopped once the target participant number was reached. Participants received oral and written information on the goal, duration and location of the FGs. They did not receive any compensation for participation. Participants were not known from the study team.

#### Clinicians

Clinician recruitment was done by e-mail by the senior author. Since the authors were also recruiting participants for a survey, an e-mail was sent to all clinicians working on the medical wards of the participating hospitals, and the first persons answering were planned for participation to the FGs until target number of participants was reached. We planned FGs with a mix of professions (one FG with two physicians, two nurses/nursing assistants and one physiotherapist in each hospital) to provide multidisciplinary insight. In addition, because we supposed some clinicians might be reluctant to express their perspectives in front of clinicians with another role (e.g., physiotherapists in front of nurses), we also planned FGs with the three professions separately (five participants in each FG) in the two largest hospitals. Participants were not chosen based on any relationship with the authors. However, some participants were known from the attending physician (CA).

### Data collection

FGs with patients who had been hospitalized in Bern University Hospital or in Tiefenau Hospital, as well as with clinicians from Bern University Hospital, were conducted at that Bern University Hospital. FGs with clinicians from Tiefenau Hospital were conducted in that hospital. FGs with patients who had been hospitalized at Fribourg Cantonal Hospital and with clinicians working at that hospital were conducted at Fribourg Cantonal Hospital, except for one FG with physiotherapists that was conducted virtually for practical reasons. The FGs were led by one author (CA or MM), with the presence of at least one co-author (CA, MM, CM) who could participate in the discussion and took field notes. No other person was present besides the participants and the researchers. FG discussions were conducted based on semi-structured guides (presented in Tables 1 and 2) that started with an introduction on the topic, followed by open-ended questions exploring mobility experiences, expectations, barriers and facilitators to mobility, consequences of low mobility, and knowledge about mobility. The discussion guides included main questions, as well as sub-questions or hints that were used to guide the participants only in case they had difficulties expressing their perspectives. All FGs were recorded and transcribed verbatim in an anonymous way. Participants were not asked for feedback on the transcripts or on the findings.

**Table 1:** Discussion guide for patient FGs

**Explorative questions: Experience & expectations of hospital stay**
**1a) Please tell us about your experience regarding mobility during your stay on the medical ward. How was it for you to move during your hospital stay?**
- Which positive or negative experiences did you have?
- Which feelings do you associate with moving during hospitalization?
- What were your doubts?
- How did you experience the help you received from the healthcare professionals? Was it important for you to move?
**1b) During your hospital stay, were you able to move according to your expectations?**
*Indications: Walking aid, family / visit support, physiotherapy, clinician availability, explanations, medical devices / tubes, room / hospital environment encouraging mobility, goal / motivation (e.g., autonomy preservation, discharge home), planning of medical examinations / rounds*
**Barriers and facilitators**
**2a) What do you think encouraged you to move during your hospital stay? What helped you to move?**
*Indications: Support, visits, hospital environment, room installation, mobility possibilities, examinations, …*
**3a) What prevented you from moving during your hospital stay? What made it more difficult to move?**
- Did you have doubts regarding mobility?
* Indications: clinician behavior, fears, abilities, lack of support, hospital environment, room installation, mobility possibilities, organization, …*
**3b) What should change in order to help increase mobility?**
**Consequences**
**4) What do you know about consequences on older adults of low mobility during a hospital stay?**
- *Optional: What was your personal experience?*
**Information**
**5) Could you please describe which kind of information regarding mobility and mobility possibilities you received during your hospital stay?**
- Which additional information would you have wished?
**General**
**6) Which additional recommendations do you have for us? What could we improve?**

Main questions are displayed in bold, sub-questions in normal font, and indications in italics. Sub-questions and indications were used to guide participants in case they had difficulties to express their perspectives

**Table 2:** Discussion guide for patient FGs

**Explorative questions: Experiences**
**1) What experiences do you have regarding mobilization and mobility of older patients on your ward? Tell us about your everyday practice, what comes to your mind.**
- How do you usually proceed? Is that a matter of concern on your ward? How do you experience patient mobility?
- Which feelings or doubts do you associate with mobility of older patients (security)?
*Indications: Walking aid, family / visit support, physiotherapy, sufficient / trained clinicians, room / hospital environment encouraging mobility, goal / motivation (e.g., progress recording), goals of the ward, planning of medical examinations / rounds*
**Barriers and facilitators**
**2a) What are barriers regarding mobilization and mobility of your patients?**
- What makes it difficult **for you** to ensure that your patients move as much as possible?
**2b) How do you see clinician responsibilities regarding patient mobility?**
**2c) What should change?** *Indications: Facilitators, organization, hospital installation, planning of *
*medical examinations / rounds, communication / documentation, patient reachability, access to walking aids, mobility-encouraging environment / room installation, …*
**3) What does facilitate mobilization and mobility of your patients?**
- What does make it easier **for you** to ensure that your patients move as much as possible? *Indications: Facilitators, organization, hospital installation, planning of *
*medical examinations / rounds, communication / documentation, patient reachability, access to walking aids, mobility-encouraging environment / room installation, …*
**4) What do you think about information available on patient mobility on your ward, for you and for your patients?**
- What could be improved?
**Consequences**
**5) What do you know about potential consequences of low hospital mobility for older patients?**
**General**
**6) Which additional recommendations do you have for us? What else do you want to communicate us?**

Main questions are displayed in bold, sub-questions in normal font, and indications in italics. Sub-questions and indications were used to guide participants in case they had difficulties to express their perspectives

### Data analysis

We applied an inductive thematic content analysis according to the six steps of Clarke and Braun [15]. The analyses were conducted based on the original data, and then translated into English by CA (who is French-speaking native, has been working for the past 10 years in German and English, and lived for two years in the USA) for publication. The translations were discussed between all authors for clarity and checked for accuracy by BM who lived for 10 years in the USA. The FGs conducted in German were coded by one author (MM). A first discussion with two senior authors (CA and JI) was conducted after initial coding of two patient FGs and two clinician FGs. Afterwards, an iterative process with discussions between those three authors was used until consensus was reached for all FGs. The FGs conducted in French were then coded by another author (CA) based on the results from the FGs conducted in German. Additional codes that raised from those FGs were discussed with MM. The third opinion of JI was sought in case CA and MM could not reach consensus. We aimed to create a framework with themes, subthemes and codes, that might require different approaches to be addressed in order to improve mobility, based on clinical reflection. Patient and clinician FGs were first coded separately. Results where then compared and wording adapted whenever possible to create similar themes, subthemes and codes between patients and clinicians. Data analysis was performed with MAXQDA software.

We use the following abbreviations to report participant citations in the article: FG, focus group; MD, physician; N, nurse; P, patient; PT, physiotherapist.

## Results

In March–April 2022, we conducted five FGs with 24 patients (4–6 patients in each group) and eight FGs with 34 clinicians (15 physicians, nine nurses/nursing assistants, 10 physiotherapists). Of 778 patients screened, 352 (45.2%) were called, while 462 (59.4%) did not meet the eligibility criteria. Among the 352 eligible patients, 30 (8.5%) accepted to participate. Among them, three (10.0%) did not attend because of acute sickness and three (10.0%) without giving a reason. Of 38 clinicians recruited, two did not attend because of acute sickness and two without giving a reason. Three FGs included a mix of professions, one FG only nursing staff, two FGs only physicians, and two FGs only physiotherapists. Characteristics of FG participants are presented in Table 3. Due to staff shortage, one FG with nurses/nursing assistants could not be organized in Fribourg Cantonal Hospital, leading to eight instead of nine FGs. Mean patient age was 71 (SD 7) years, with 10 (42%) women. The authors did not identify new codes after analyzing 3/5 (60%) of patient and 6/8 (75%) of clinician FGs. They thus estimated having reached data saturation and did not plan additional FGs.

**Table 3:** Participant characteristics

**Variable**	**Distribution**
Patients	
Age, median (minimum–maximum)	69 (61–86)
Female, N(%)	10 (41.2%)
Length of stay, median (minimum–maximum)	7 (2–22)
Hospital, N(%)	
Bern University Hospital	10 (41.2%)
Tiefenau Hospital	3 (12.5%)
Fribourg Cantonal Hospital	11 (45.8%)
Main hospitalization reason	
Cardiovascular disorder	9 (37.5)
Infectious disease	7 (29.2)
Gastroenterological disorder	2 (8.3)
Neurological disorder (other than stroke)	2 (8.3)
Diabetes decompensation	1 (4.2)
Other	3 (12.5)
Clinicians	
Profession, N(%)	
Nursing staff	9 (26.5%)
Physician	15 (44.1%)
Physiotherapist	10 (29.4%)
Female, N(%)	22 (64.7%)
Hospital, N(%)	
Bern University Hospital	16 (47.0%)
Tiefenau Hospital	4 (11.8%)
Fribourg Cantonal Hospital	14 (41.2%)

**Fig. 1:**
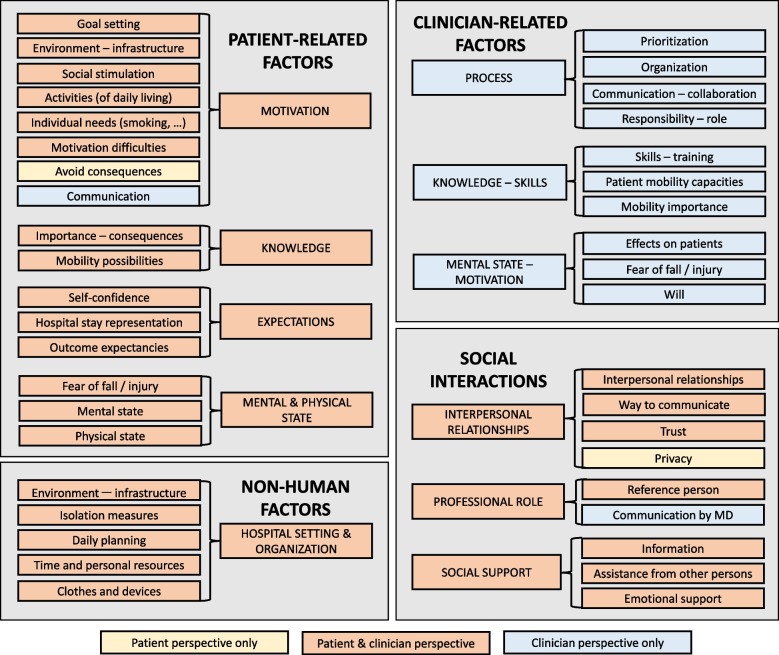
Framework displaying themes, subthemes and codes obtained through the thematic analysis Legend: The four themes (patient-related factors, clinician-related factors, social interactions, non-human factors) are displayed in capital letters in the large grey boxes. Subthemes are displayed in capital letters, and codes in lower case, in the colored boxes. Subthemes and codes in coral boxes come from both patient & clinician FGs, those in yellow boxes from patient FGs only, and those in blue boxes from clinician FGs only. Abbreviations: MD, medical doctor

We identified four themes of barriers and facilitators to mobility: 1) patient-related factors; 2) clinician-related factors; 3) social interactions; and 4) non-human factors. Each theme included 1–4 subthemes with 2–8 codes. Figure 1 presents the framework obtained through the thematic analysis, displaying themes, subthemes and codes. Additional file 1: Table 1 presents theme development from patient perspective, and Additional file 1: Table 2 from that of clinicians.

### Theme 1: patient-related factors

#### Motivation

Patient motivation was perceived as a determinant of mobility by patients and clinicians. Setting not only specific in-hospital goals (e.g., walking to the cafeteria), but also patient-relevant goals, such as returning home, was seen as a facilitator. However, some patients felt pressured by goals set by clinicians without consulting them (FG1P2): *“It is a little bit this pressure from physiotherapists: “And now! My goal is, today, you get up, you come with me, we make a round.” Yes, this is her opinion, but I also have one.”*

Some factors in the environment and infrastructure, such as the possibility to go outside or have access to a room with books or TV were seen as motivators, while the hospital environment was described as unattractive (FG3P4): *“But yes, it could be attractive if there was something to look at in the corridor. Because it is quite sober. I mean, there is nothing!”.*

Social stimulation though hospital staff and relatives was seen as a motivator (FG3P2): *“I was well lying in bed, but my husband told me, but move, move, look what you can do at home. It was him seeing that I was not doing well, because, finally, ourselves, maybe we don’t realize.”* FG9N4:* “And the relatives, I think, are motivators.”*

Activities of daily living were used to motivate patients (FG13PT2): *“Trying different subterfuges to take them out of this state, for example dressing them, putting the meal on the table without asking if they want to eat in bed or at the table. It brings a form of naturalness.”* Patients described what could motivate them to move in their activities of daily living (FG5P3): “*Eating in front of the view from the sixth floor, it’s wonderful. … That’s already a way to be mobile. Even if this is all we can do.”*

Individual needs (e.g., to move, going for a smoke) were described as motivators (FG6MD1): *“The biggest motivation is smoking.”* But both clinicians and patients also expressed motivation difficulties, such as patients lacking the desire to move or enjoying eating in bed (FG3P2): *“I could have eaten sitting in the armchair. Just going out of bed and sitting in the armchair, even if it is next to the bed, it is already something. But no, I found it so much more comfortable to eat in bed!”.*

Patients said they wanted to move to avoid adverse consequences (FG2P3): *“Because I needed to stay independent and go back home as soon as possible and be functional there independently without home care.”*

Clinicians felt what they communicated to the patients could influence their motivation (FG6N1): *“Yes, we tell them [the patients], that it is important to get up, that they lose muscle every day when they stay in bed. Yes, also what it can have as consequences. That they also want to go back home. To motivate.”*

#### Knowledge

This subtheme included factual knowledge (consequences of low mobility) and action knowledge (if, where, or how to move). Regarding factual knowledge, patients could cite short-term, but not long-term complications of low mobility. This lack of factual knowledge was noticed by clinicians, who emphasized the importance of education. Regarding action knowledge, patients reported they did not know if, where, or how they were allowed to move. This was similarly perceived by clinicians (FG11MD1): *“Sometimes, people look at us with wide eyes when we say: “But you should go to the terrace to take some fresh air!” – “Ah, but am I allowed to?””.*

#### Expectations

Clinicians perceived a pervasive “bedrest mindset” or “sick-role behavior” among patients and relatives, expressed by patients who expected to rest rather than to move around (FG11MD2): *“But I think, many patients kind of have this idea, I am in a hospital, I have to stay in bed”.*

Clinicians expressed a need to address patient lack of self-confidence and false expectations (FG7MD2): *“Because when they expect it [return to normal capacities] will be perfect within two days, they do not do anything anymore. And I think we say that too rarely. That sometimes, it takes time.”*

#### Mental and physical state

Another barrier was the fear of falling (FG1P1): *“I was so often lying down, and for me it was always … The fear of standing up… Yes, the fear of injury”*. Other barriers included mental issues, such as indifference (FG1P1): *“It was a kind of indifference. Whether I was now leaving or dying, whether it is going well or bad, I didn’t care…”* And physical state (FG3P2): *“Not enough strength. It is not that I did not want to, but after a few days, I couldn’t handle it anymore.”*

### Theme 2: clinician-related factors

#### Process

Clinicians identified the lack of systematic process as a barrier. They suggested organizational modifications, such as asking patients to bring back their pillboxes or to walk to another room for medical rounds. They expressed not prioritizing mobility enough, despite recognizing its importance (FG9N1): *“But I think nurses, when prioritizing, it is typical, conversations and mobility, they are de-prioritized first. And I think, here other things could be dropped.”*

Fall risk assessment was perceived as having advantages and disadvantages: *“It is a little bit double-edged, because we can interpret that [the risk of fall] as a prohibition to move, because it [moving] is dangerous. But actually, what it [fall risk assessment] should be used for, is to be careful about the risk of falling, and therefore to mobilize patients. Not to not mobilize them.”*

Clinicians also mentioned a lack of communication and structured interdisciplinary collaboration (FG12PT2): *“I got feedback from a nurse, and I hoped the nurse would tell the physician during the rounds “and he [the patient] walked up the stairs without a physiotherapist”. But I cannot control for that. And if I had a way to tell such things directly to the physicians once a day or three times a week, a short moment, that all disciplines are updated, it would be great.”*

Roles and responsibility of clinicians regarding mobility were also discussed (FG12PT1): *“Because I think, there are wards where it is clear that mobilization is a physiotherapist task, and there are other wards where it is absolutely a shared task of nurses and physiotherapists.”*

#### Knowledge – skills

Physiotherapists highlighted a lack of nurses’ skills, which they addressed through bedside teaching (FG7PT1): *“It is actually a big part of everyday practice for nurses and they are not trained. And I find it is a pity.”* Physicians and nurses acknowledged they lacked specific training on mobility, while physicians mentioned not being aware of patient mobility capacities (FG11MD2): *“Because you see, often, the patient goes to the cafeteria with his family, or upstairs, or something like that, and you don’t always see him. And sometimes I felt, this patient doesn’t move at all. And when I asked him, “but yes, yesterday I went for a walk…” And that’s just a moment where you were not there, that’s it, you didn’t see him.”*

Clinicians were aware of the consequences of low mobility (FG10MD1): *“Unfortunately, it is frequent … that a patient lies for days and is even not mobilized at bedside. Complications that we have to accept, are considerable.”*

#### Mental state – motivation

Not only patient fear of falling, but also clinician fear of patient injury, were mentioned. Systematic fall risk assessment was found to strengthen this (FG8N1): *“What I have noticed, it is that since we are screening [for fall risk], patients move even less, because we are afraid.”* Clinicians were aware their stress could negatively impact the patients (FG7N2): *“So I noticed, when I am highly stressed, then I transmit that also to the patients.”* Physiotherapists felt nurses were not always motivated or willing to mobilize patients (mentioned in FG with only physiotherapists).

### Theme 3: social interactions

#### Interpersonal relationships

Interpersonal relationships between patients and between patients and clinicians were noted as important to build trust and motivation (FG13PT4): *“First, they [the patients] need to trust us. … I try to let them express their fears. And then, when I feel there is a trusting relationship … This can be done while walking.”* Patients appreciated one-on-one conversations with clinicians, but expressed privacy concerns preventing mobility, e.g., feeling uncomfortable hearing about other patients or seeing into other patients’ rooms when walking in the corridors (FG4P2): *“Uncomfortable because the room doors are open … so we see things we should not be seeing.”*

Trust was also expressed as important (FG1P1): *“When someone takes a moment to sit with you, …it builds trust and allows you to listen to what they are saying.”*

#### Professional role

Nurses and physiotherapists thought it would be helpful if physicians were more involved, because patients might trust them more (FG3N1): *“If the physicians would say, it is good to stand up and move as much as possible, it might help, because patients really feel physicians know more, so they trust the physician.”* But clinicians perceived nurses as reference persons to best motivate patients (FG12PT4): *“We experience often that the nurse is the first reference person who can also strongly and best motivate the patients.”* Conversely, what mattered to patients was not the clinician involved, but that they take time for them (FG1P1): *"When a person takes time to sit beside you, no matter who it is….”*

#### Social support

Patients said they lacked information on mobilization (FG4P3): *“If you are told, “Yes, you should walk,” ok, but the goal of walking and which possibilities.”* Clinicians believed informing the patients and their relatives on the consequences of low mobility was important to increase motivation.

Patients mentioned that being accompanied helped them feel safe. Clinicians thought and wished relatives and volunteers could help patients (FG6MD1): *“When all it is about is that people move, then it doesn’t need highly-specialized staff… Relatives could do that as well.”*

Emotional support, such as empathy and encouragements, was felt to increase motivation and self-confidence (FG2P1): *“I can remember how one day I walked in the corridor with the walking aid, it was endless and when I turned around, they [the clinicians] were all there and clapped. And said “Wow, you did it….” And it was such a relief to know, wow, you can do it until there and you are never alone.”*

### Theme 4: non-human factors

#### Hospital setting and organization

Patients and clinicians identified several environmental barriers, such as lack of seating or space in the corridors/rooms (FG11MD2): *“Even in the corridor, there is no space.”* FG11MD1 answering *“Yes, there are carts everywhere, really, it’s dangerous!”* Clinicians suggested modifying the environment to foster mobility (e.g., coffee/book corner, handrails and mobility incentives in corridors, folding beds). Isolation due to COVID was mentioned as a barrier (FG11MD1): *“We no longer put the patients at the table for eating, so people stayed in bed for eating.”*

Another barrier was the lack of schedule. Clinicians wished they could give time slots to patients, while patients stayed in room to avoid missing medical rounds or an examination (FG2P2): *“… I never knew when I would have an examination … I almost always had to stay in room because it could then mean anytime, “Mister X, you have to come immediately, we have a slot for you for the CT-scan.”*

Clinicians unanimously said they needed more staff and time to manage patient mobility, while patients were reluctant to seek support when perceiving clinician workload (FG1P4): *“We see how the nursing staff is busy. And then, I have, most often we have, we don’t dare to say anything.”*

Hospital gowns and devices such as tubing or catheters were preventing mobility, while wearing one’s own clothing was facilitating it (FG13PT2): *“As soon as patients can, that there is no more invasive exam requiring an easy access to the body, we encourage them, we help them to put on their sweatpants, their clothes.”*

## Discussion

In this qualitative study, we assessed patients’ and clinicians’ perspectives on older patients’ mobility during an acute medical hospitalization. Subthemes from patient and clinician FGs were similar, except that clinician-related factors were identified in clinician FGs only. Codes were mostly overlapping. New factors identified in this study include privacy issues and role perception. Many barriers and facilitators are potentially actionable in everyday clinical practice, even without requiring additional resources.

While most aspects mentioned by patients were also reflected by clinicians, suggesting that the latter perceive patient situation accurately and consistently, three discrepancies are worth discussing. First, patients expressed privacy issues preventing mobility not mentioned by clinicians (e.g., seeing into other patients’ rooms when ambulating). Clinicians must be aware of this barrier that seems rather easy to address, e.g., by discussing patients’ situation in patient rooms with closed doors during the medical rounds, or in nursing staff or physician office, but not in the corridors. Second, whereas clinicians from all three professions perceived nurses as reference persons to motivate the patients, patients did not care about the profession of the clinician involved. Previous studies identified clinician responsibilities/roles regarding mobility, but did not highlight such discrepancy between patient and clinician perspectives [11, 16–22]. This should be considered during clinician training and when defining roles and processes regarding hospital mobility. Third, patients did not mention clinician-related factors. Educating patients on clinician-related factors, such as their skills, roles and responsibilities, could be help patients ask for clinician support.

Lack of time and staff resources were mentioned frequently, consistent with previous findings [11, 16, 23]. Patients perceiving clinician stress and lack of time did not dare asking for help. However, while adding staff resources improved mobility in studies [8, 9, 24–26], it is unlikely to modify practices in a context of staff shortage and rising healthcare costs. Conversely, focusing on optimizing the use of time and staff resources, for example by clarifying staff roles, ways to communicate and mobility task planning, could be a way to address these barriers. Clinicians indeed highlighted the lack of standardized process regarding hospital mobility, including role definition, communication and documentation. While other elements of care process, such as bowel movement, are systematically documented and consecutively discussed between clinicians, the same does not apply to mobility. Mobility was a nursing responsibility previously, but seems to have been partly removed from nursing tasks and training, and shifted to physiotherapists only [27, 28]. Systematizing and standardizing processes regarding hospital mobility, and ensuring physician and nurse training, could help prioritize mobility.

Patients’ representations preventing mobility (“stay in bed when sick”), mentioned by patients and clinicians, are consistent with previous data [11, 16, 22, 23]. In addition, we identified a lack of patient knowledge on whether, when, how and where to move, which was not obvious in previous works. Clinicians should thus not take for granted that patients know that. Conversely, they should provide patients with concrete information (e.g., where is the cafeteria, at what time they can go there). Education, effective in previous studies [29, 30], should thus be part of future interventions and target not only patients, but also their relatives and friends who can promote mobility and also seem to lack education on hospital mobility [31].

Our work outlines several factors that can facilitate mobility. First, defining concrete and individualized not only short-term (e.g., eating at the table), but also long-term (e.g., returning home) mobility goals with the patients, could help improve mobility. Second, modifying the hospital environment, identified by patients and clinicians in our study and in previous works as unstimulating and boring [11, 16, 32, 33], could encourage patients to move more; for example, hanging on posters or pictures on topics of interests to the patients to make the ward more patient-friendly. Finally, as short hospitalizations and early mobilization become the rule, it might be time to rethink the hospital in and of itself, a concept that has barely changed in the last decades. Innovations to fight the epidemic of low hospital mobility could include folding hospital beds, common dining rooms, patients having to retrieve their medications from the nurses, or conducting medical rounds in a separate room. Developing the concept of hospital-at-home might be an additional way to improve mobility during an acute illness [34]. Such changes are not completely unrealistic, but imply considerable changes in mindsets, behaviors and practices, and would therefore require the involvement of higher-level health and policy makers.

### Limitations and strengths

This study has several strengths. First, we included both patients and clinicians, who are key stakeholders of hospital mobility. Second, we conducted FGs mixing and separating professions, which identified some issues that were not expressed in mixed FGs. For example, physiotherapists expressed lack of training in mobility observed among nursing staff. Third, we estimated we had reached saturation with our data. Finally, we used an iterative coding process with three authors.

We must acknowledge some limitations. First, this study was conducted in Switzerland only. However, we covered different language/cultural regions and hospital sizes, and considering local context is recommended in implementation science [35]. Second, we did not assess the perspectives of relatives and friends who can influence patient mobility. Third, we cannot exclude a risk of bias, since patients and clinicians who agreed to participate might have been particularly motivated or interested by the topic. However, making participation mandatory would not have been possible for ethical reasons. Finally, the inductive thematic analysis not based on an existing framework might limit comparison with other studies. However, most subthemes identified are also found in various theoretical frameworks (e.g., Theoretical Domains Framework) [36].

## Conclusion and implications

Our study integrating patients’ and clinicians’ perspectives on mobility during an acute medical hospitalization provides a practical framework that can be applied in clinical practice to improve mobility of older hospitalized patients. This study identified new factors of hospital mobility, such as privacy issues and role perception, and extends previous knowledge. It is a first step towards participatory research, providing key information on factors related to mobility in this context. Our findings can help clinicians and researchers identify actionable barriers and facilitators and thus find concrete solutions to improve hospital mobility. To successfully change practices, future studies and quality improvement initiatives should be participative and involve all stakeholders of hospital mobility to ensure they address barriers and facilitators in a way that suit their needs on the long term and in real life.

### Supplementary Information

**Additional file 1: Appendix Table 1. **Thematic analysis of patient perspectives. **Appendix Table 2. **Thematic analysis of clinician perspectives.

## Data Availability

Coding and transcripts are available from the corresponding author upon reasonable request.

## Abbreviations

FG: Focus group
MD: Physician
N: Nurse
P: Patient
PT: Physiotherapist
