# Supplementary material for: Perspectives of patients and clinicians on older patient mobility on acute medical wards: a qualitative study
Source: BMC Geriatr. 2023 Sep 13;23:558. doi: 10.1186/s12877-023-04226-0 (PMC10500927; doi:10.1186/s12877-023-04226-0)
Supplement: Supplementary file 1 — Additional file 1: Appendix Table 1. Thematic analysis of patient perspectives. Appendix Table 2. Thematic analysis of clinician perspectives. [file 12877_2023_4226_MOESM1_ESM.docx]

Perspectives of patients and clinicians on older patient mobility on acute medical wards: a qualitative study

**SUPPLEMENTARY FILE**

**Appendix Table 1.** Thematic analysis of patient perspectives.

| **THEMES** | **SUBTHEMES** | **CODES** |
| --- | --- | --- |
| PATIENT-RELATED FACTORS | MOTIVATION | Goal setting |
|  |  | Environment – infrastructure |
|  |  | Social stimulation |
|  |  | Activities (of daily living) |
|  |  | Individual needs (smoking, …) |
|  |  | Motivation difficulties |
|  |  | Avoid consequences |
|  | KNOWLEDGE | Importance – consequences |
|  |  | Moving possibilities |
|  | EXPECTATIONS | Self-confidence |
|  |  | Hospital stay representation |
|  |  | Outcome expectancies |
|  | MENTAL AND PHYSICAL CONDITION | Fear of fall / injury |
|  |  | Mental state |
|  |  | Physical state |
| SOCIAL  INTERACTIONS | INTERPERSONAL RELATIONSHIPS | Interpersonal relationships |
|  |  | Way to communicate |
|  |  | Trust |
|  |  | Privacy |
|  | PROFESSIONAL ROLE | Reference person |
|  | SOCIAL SUPPORT | Information |
|  |  | Assistance from other persons |
|  |  | Emotional support |
| NON-HUMAN FACTORS | HOSPITAL SETTING AND ORGANIZATION | Environment – infrastructure |
|  |  | Isolation measures |
|  |  | Daily planning |
|  |  | Time and staff resources |
|  |  | Clothes and devices |

**Appendix Table 2.** Thematic analysis of clinician perspectives.

| **THEMES** | **SUBTHEMES** | **CODES** | |
| --- | --- | --- | --- |
| PATIENT-RELATED FACTORS | MOTIVATION | | Goal setting |
|  |  |  | Environment/infrastructure |
|  |  |  | Social stimulation |
|  |  |  | Activities of daily living |
|  |  |  | Individual needs (smoking, …) |
|  |  |  | Motivation difficulties |
|  |  |  | Communication |
|  | KNOWLEDGE | | Importance – consequences |
|  |  |  | Mobility possibilities |
|  | EXPECTATIONS | | Self-confidence |
|  |  |  | Hospital stay representation |
|  |  |  | Outcome expectancies |
|  | MENTAL AND PHYSICAL STATE | | Fear of fall – injury |
|  |  |  | Mental state |
|  |  |  | Physical state |
| CLINICIAN-RELATED FACTORS | PROCESS | | Prioritization |
|  |  |  | Organization |
|  |  |  | Communication – collaboration |
|  |  |  | Responsibility – role |
|  | KNOWLEDGE – SKILLS | | Skills - training |
|  |  |  | Patient mobility capacities |
|  |  |  | Mobility importance |
|  | MENTAL STATE – MOTIVATION | | Effect on patients |
|  |  |  | Fear of fall / injury |
|  |  |  | Will |
| SOCIAL INTERACTIONS | INTERPERSONAL RELATIONSHIPS | | Interpersonal relationships |
|  |  |  | Way to communicate |
|  |  |  | Trust |
|  | PROFESSIONAL ROLE | | Nurse as referent |
|  |  |  | Communication through physician |
|  | SOCIAL SUPPORT | | Information |
|  |  |  | Assistance from other persons |
|  |  |  | Emotional support |
| NON-HUMAN FACTORS | HOSPITAL SETTING AND ORGANIZATION | | Environment – infrastructure |
|  |  |  | Isolation measures |
|  |  |  | Daily planning |
|  |  |  | Time and staff resources |
|  |  |  | Clothes and devices |
